# Activation of oncogenic tyrosine kinase signaling promotes insulin receptor-mediated cone photoreceptor survival

**DOI:** 10.18632/oncotarget.10447

**Published:** 2016-07-06

**Authors:** Ammaji Rajala, Yuhong Wang, Raju V.S. Rajala

**Affiliations:** ^1^ Department of Ophthalmology, University of Oklahoma Health Sciences Center, Oklahoma City, OK, USA; ^2^ Department of Physiology, University of Oklahoma Health Sciences Center, Oklahoma City, OK, USA; ^3^ Department of Cell Biology, University of Oklahoma Health Sciences Center, Oklahoma City, OK, USA; ^4^ Dean McGee Eye Institute, Oklahoma City, OK, USA

**Keywords:** age-related retinal degeneration, insulin receptor, Src kinase, cone photoreceptors, tyrosine phosphorylation, Gerotarget

## Abstract

In humans, daylight vision is primarily mediated by cone photoreceptors. These cells die in age-related retinal degenerations. Prolonging the life of cones for even one decade would have an enormous beneficial effect on usable vision in an aging population. Photoreceptors are postmitotic, but shed 10% of their outer segments daily, and must synthesize the membrane and protein equivalent of a proliferating cell each day. Although activation of oncogenic tyrosine kinase and inhibition of tyrosine phosphatase signaling is known to be essential for tumor progression, the cellular regulation of this signaling in postmitotic photoreceptor cells has not been studied. In the present study, we report that a novel G-protein coupled receptor–mediated insulin receptor (IR) signaling pathway is regulated by non-receptor tyrosine kinase Src through the inhibition of protein tyrosine phosphatase IB (PTP1B). We demonstrated the functional significance of this pathway through conditional deletion of IR and PTP1B in cones, in addition to delaying the death of cones in a mouse model of cone degeneration by activating the Src. This is the first study demonstrating the molecular mechanism of a novel signaling pathway in photoreceptor cells, which provides a window of opportunity to save the dying cones in retinal degenerative diseases.

## INTRODUCTION

Photoreceptors have high metabolic activity [1], with an energy consumption equivalent to that of proliferating cancer cells [2, 3]. The difference between a photoreceptor cell and a cancer cell is that photoreceptors are non-dividing postmitotic cells, while cancer cells actively divide. However, photoreceptors shed 10% of their outer segments daily [4], they must synthesize the membrane and protein equivalent of a proliferating cell each day. Oncogenic tyrosine kinase signaling is essential for tumor progression. This signaling has not been studied in photoreceptor cells.

In humans and rodents, cone photoreceptors constitute 3-to-5 percent of retinal photoreceptor neurons [5, 6], and they are indispensable in humans for color vision, optimal visual acuity, and visual sensitivity under different light intensities. Cone photoreceptors are commonly affected in age-related macular degeneration (AMD) [7–9] and diabetic retinopathy (DR) [10]. Retinitis pigmentosa (RP) is an inherited retinal degenerative disease in which cones are secondarily affected [11, 12], whereas cones are directly affected in cone and cone-rod dystrophies [13]. Prolonging the life of foveal cones (daylight vision) for even one decade would have an enormous beneficial effect on usable vision in an aging population. Possible treatment options to preserve useful vision include gene therapy, delivery of growth factors, antioxidant therapy, stem cell therapy, nanoparticles, optogenetics, and gene-editing techniques. For example, more than 140 mutations leading to the secondary death of cones have been identified in the rhodopsin gene (www.retina-international.org); in some cases, patients may have more than one mutation. If the mutation is recessive, the gene can be replaced. If the mutation is dominant, the gene can be knocked down. It is desirable to have a universal treatment for these retinal diseases.

**Figure 1:**
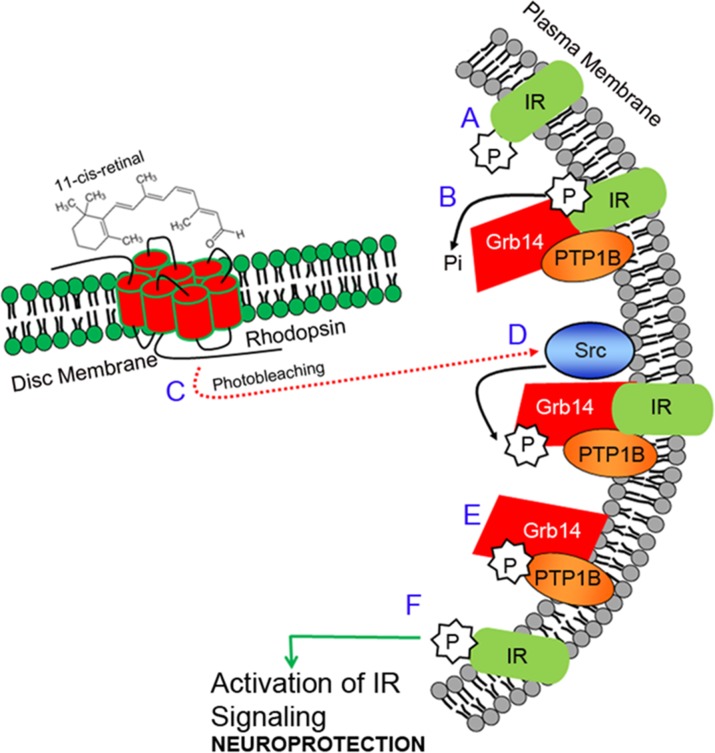
Insulin receptor signaling pathway in photoreceptors The insulin receptor (IR) in the retina is constitutively phosphorylated (activation; **A.**). Activation of the IR is negatively regulated by PTP1B and Grb14. PTP1B directly dephosphorylates phosphate groups on the IR, while Grb14 directly binds to the active site of the IR and inactivates it **B.**. The IR overcomes the inactivation by both PTP1B and Grb14 through the photobleaching of rhodopsin **C.**, which activates a non-receptor tyrosine kinase, Src, by an unknown mechanism **D.**. Src phosphorylates Grb14, and the phosphorylated Grb14 binds to the active site of the PTP1B and inactivates it **E.**, thereby relieving the inhibitory constraints on the IR **F.**. Thus, the IR becomes active, and regulates the downstream effector cascade, which provides neuroprotection to the retina. IR, insulin receptor; P, phosphorylation; Pi, inorganic phosphate; Grb14, growth factor receptor bound-protein 14; Src, non-receptor tyrosine kinase Src; PTP1B, protein tyrosine phosphatase 1B.

We discovered a novel light-activation of the insulin receptor (IR) signaling pathway in retinal rod photoreceptor neurons (Figure 1). Insulin receptors are confined to the plasma membrane of outer and inner segments of both rod and cone photoreceptor neurons [14, 15]. Interestingly, in the retina, the IR has a high basal activity (phosphorylation), presumably due to the lack of exon 11 in the extracellular domain [16, 17]. However, in the dark, this activity is negatively regulated by protein tyrosine phosphatase, PTP1B [18], and growth factor receptor-bound protein 14 (Grb14) [19]. The former protein is a phosphatase and the latter is an adapter protein. The PTP1B dephosphorylates IR, and the un-phosphorylated form of Grb14 inactivates IR by direct binding to the catalytic loop of the IR; consequently, the IR becomes inactive [20, 21]. In light, the IR overcomes the inactivation by PTP1B and Grb14 through photobleaching of rhodopsin, which activates a non-receptor tyrosine kinase, Src [22, 23], that phosphorylates Grb14. The phosphorylated Grb14 acts a competitive inhibitor of PTP1B and inhibits its activity [22], and protects the IR from dephosphorylation [23]. The activated form of IR regulates the neuroprotective downstream effector cascade. Thus, IR signaling is temporarily and spatially regulated through activation and deactivation of proteins under dark- and light-adapted conditions. In the present study, we examined this tyrosine kinase signaling pathway in cones, and further activated this pathway genetically and therapeutically in retinal degeneration to promote cone survival. We demonstrated the functional significance of this pathway through conditional deletion of IR and PTP1B in cones, in addition to delaying the death of cones in a mouse model of cone degeneration, by activating the Src. This is the first study demonstrating the molecular mechanism of a novel signaling pathway in photoreceptor cells, which provides a window of opportunity to save the dying cones in retinal degenerative diseases.

## RESULTS

### Light-dependent association of the inulin receptor with phosphoinositol 3-kinase (PI3K)

**Figure 2:**
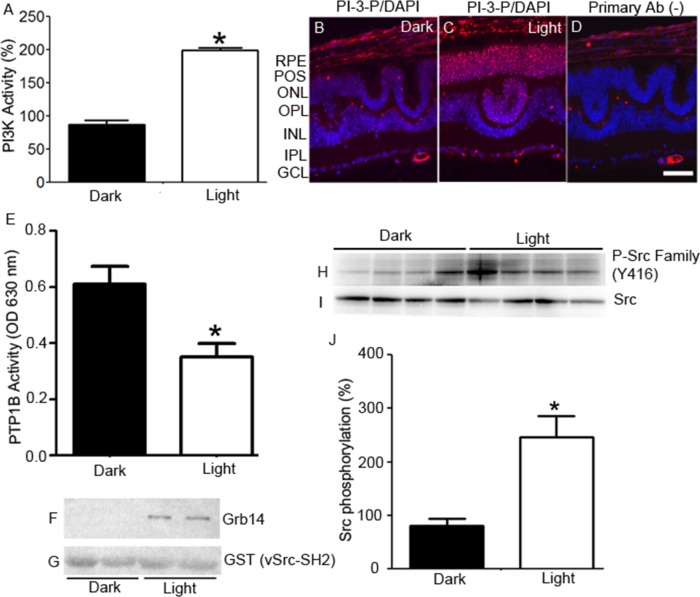
Light-dependent regulation of IR signaling proteins in cones Retinal lysates from dark- and light-adapted (300 lux, 30 min) *Nrl^−/−^* mice were immunoprecipitated with anti-IR antibody, and measured for PI3K activity using PI-4,5-P_2_ and [γ^32^P]ATP as substrates. The radioactive spots of PI-3,4,5-P_3_ were scraped from the TLC plate and counted **A.**. Data are means + *SE* (*n* = 4). *P < 0.001. The dark-adapted control (insulin receptor-associated PI3K activity) was set at 100%. Prefer-fixed sections of dark- **B.** and light-adapted **C.** *Nrl^−/−^* mouse retinas were stained for PI-3-P (red) and DAPI (blue). Panel **D.** represents the omission of PI-3-P antibody. RPE, retinal pigment epithelium; POS, photoreceptor outer segments; ONL, outer nuclear layer; OPL, outer plexiform layer; INL, inner nuclear layer; IPL, inner plexiform layer; GCL, ganglion cell layer. PTP1B activity was measured in the retinas harvested from the dark- and light-adapted *Nrl^−/−^* mice **E.**. Data are means +*SE*, *n* = 5, *P < 0.05. Dark- and light-adapted *Nrl^−/−^* mouse retinal lysates (duplicate) were incubated with GST-vSrc-SH2 fusion protein, followed by GST pull-down assay. The bound proteins were immunoblotted with anti-Grb14 **F.** and anti-GST **G.** antibodies. Retinas from four dark- and light-adapted *Nrl^−/−^* mice were immunoblotted with P-Src family (Y416) **H.** and Src **I.** antibodies. Quantified data (phosphorylated Src normalized to total Src) are means ± *SE* (*n* = 4), *p < 0.05 **J.**. The dark-adapted control was set at 100%.

Retinal lysates from dark- and light-adapted cone-dominant *Nrl^−/−^* mice [15] were immunoprecipitated with anti-insulin receptor (IR) antibody and were measured for IR-associated PI3K activity using substrates of [^32^P] adenosine triphosphate and phosphoinositide-4,5-bisphosphate (PI-4,5-P_2_). The PI3K-generated PI-3,4,5-P_3_ radioactive spots were scraped from the thin-layer chromatography plate, and we counted the radioactivity (Figure 2A). These observations show a light-dependent tyrosine phosphorylation of IR, which results in the association with its downstream effector, PI3K, in cones.

### *In vivo* light-dependent generation of PI-3-P

The lipid substrate phosphatidylinositol (PI) serves as a substrate for PI3K to form PI-3-P [24]. PI-3-P antibody was used to stain retinal sections from dark- and light-adapted *Nrl^−/−^* mice. We found increased generation of PI-3-P in light-adapted retina, but not in dark-adapted retina (Figure 2B-2D). These observations suggest an active, light-induced, PI3K-mediated generation of phosphoinositide second messengers in cones.

### Light regulates the PTP1B activity in cones *in vivo*

Retinal lysates from dark- and light-adapted *Nrl^−/−^* mice were subjected immunoprecipitation with anti-PTP1B antibody, and we then measured the phosphatase activity [22]. We found significantly increased PTP1B activity in dark-adapted retinas compared with light-adapted retinas (Figure 2E). These observations suggest that PTP1B phosphatase activity is inhibited by light in cones *in vivo.*

### Light-dependent tyrosine phosphorylation of Grb14 in cone-dominant retina

We previously reported that the SH2-domain of vSrc interacts with phosphorylated Grb14 *in vitro* [23]. We used this probe to determine the *in vivo* phosphorylation of Grb14 in cone-dominant retina. We incubated vSrc-SH2 fusion proteins with dark- and light-adapted *Nrl^−/−^* mouse retinal lysates. Bound proteins were recovered by a GST pull-down assay. Immunoblots were run and probed with anti-Grb14 (Figure 2F) and anti-GST (Figure 2G) antibodies. The GST blot ensured an equal amount of fusion in each GST pull-down assay. We found the interaction of Grb14 with v-SH2 domain in light-adapted conditions, but not in dark-adapted conditions (Figure 2F), suggesting that tyrosine phosphorylation of Grb14 is light dependent in cones.

### Light-dependent tyrosine phosphorylation of Src in the cone-dominant retina

Retinal lysates from dark- and light-adapted *Nrl^−/−^* mice were subjected to immunoblot analysis with anti-phospho-Src-family (Y416) and anti-Src antibodies. We found an enhanced tyrosine phosphorylation of Src in light-adapted retinas compared with dark-adapted retinas (Figure H-J). These observations suggest that Src undergoes a light-dependent tyrosine phosphorylation, which shows the activated state of Src in cone-dominant retina

### Photobleaching of opsin is required for the activation of the IR to provide survival signaling in cones

**Figure 3:**
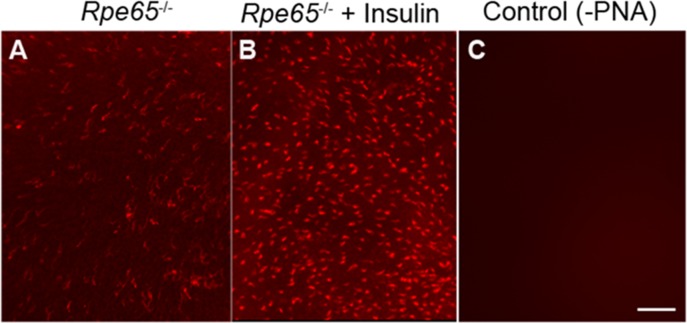
Insulin delays the death of cone photoreceptors in *Rpe65^−/−^* mice PNA-stained retinal flat mounts from 4-week-old untreated *Rpe65^−/−^* mice **A.** and *Rpe65^−/−^* mice treated (starting at P5) with daily intraperitoneal injections of insulin for 3 weeks **B.**. Control: Omission of PNA **C.**. Scale bar 100 μm.

We previously reported that, in *Rpe65*^−/−^ mice, the IR phosphorylation was abolished [14]. In this mouse model, cones start to degenerate at two weeks of age. By four weeks of age, a substantial loss of cone cells is seen in large areas of the central retina [25]. These mice do not show a rod ERG response [26], but do show a minute cone ERG response, due to isorhodopsin [27, 28]. These findings led to the hypothesis that, the normal light-driven activation of IR signaling in cones does not occur in the absence of cone-opsin bleaching, resulting in cone degeneration. To test this hypothesis, starting at postnatal day 5 (P5), we intraperitoneally injected *Rpe65^−/−^* mice with insulin daily for three weeks. At four weeks, mice were killed by CO_2_ asphyxiation. Retinal flat mounts were prepared. We examined the cone cell loss using peanut agglutinin staining (PNA). Our results suggest a delay of cone cell death in insulin-injected retinas, whereas untreated retinas lost almost all cones by four weeks (Figure 3). The absence of photobleaching is known to affect phototransduction, cone opsin mislocalization, and arrestin trafficking, but our experiments for the first time suggest that photobleaching of cone opsin may be essential to activate the IR survival signaling pathway in cones.

### Generation of cone conditional IR knockout mice

**Figure 4:**
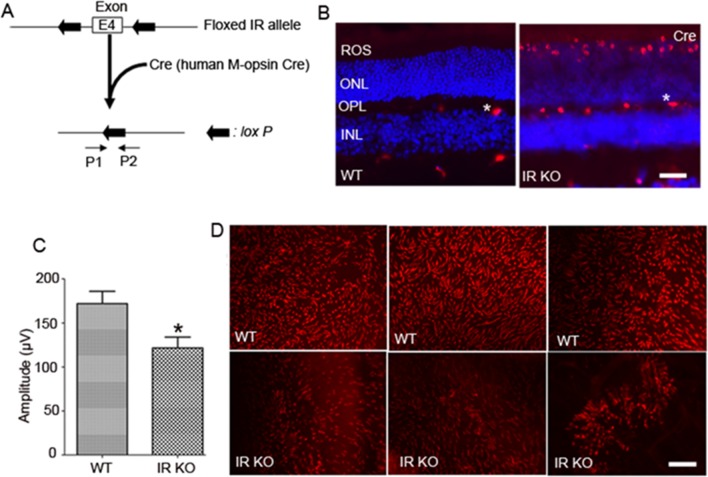
Cone-specific deletion of the IR in WT mice leads to cone degeneration Schematic diagram of *loxP* floxed IR loci **A.**. Cone photoreceptor-specific IR knockout mice were generated by breeding mice with a floxed IR with mice that express Cre recombinase under the control of human M-opsin promoter. Primer pairs P1 and P2 were used to identify the wild-type and the floxed IR alleles. Immunohistochemical analysis of Cre recombinase in wild-type and IR KO retinas **B.**. ROS, rod outer segments; ONL, outer nuclear layer; OPL, outer plexiform layer; INL, inner nuclear layer. Labeling of blood vessels (*) is non-specific. Photopic b-wave **C.** electroretinographic (ERG) analysis of WT and IR KO mice at 6 months of age. Values are means ± *SEM*. * *P* < 0.05 (*n* = 10). Retinal flat mounts from three independent 6-month-old WT littermates and cone-IR KO mouse **D.** retinas were stained with PNA. Scale bar 100 μm.

At birth, mice exhibit no signs of global deletion of IRs, but die from ketoacidosis due to early postnatal diabetes [29, 30]. To evade these potential barriers, cone conditional deletion of IR was achieved using a Cre/lox technology (Figure 4A). In this strategy, M-opsin (red/green photopigment) promoter-driven Cre-recombinase mice were mated with mice expressing floxed IR allele [31]. To identify floxed mice carrying Cre-recombinase, genotyping was performed on tail DNA samples, as described earlier [31, 32]. Our laboratory has previously shown that insulin receptors are expressed in both rods [33] and cones [15]. Therefore, studying the cone-specific deletion of IR is technically challenging. Hence, we examined the cellular localization of Cre as a surrogate marker for IR deletion in wild-type and cone-IR knockout littermates by immunofluorescence. Our results show the absence of Cre in wild-type retina and the presence of Cre expression in the nuclei of cone photoreceptors (Figure 4B).

### Effect of cone IR deletion on retinal function

Electroretinography (ERG) was used to determine the effect of IR loss on retinal function in cones. Rod function was assessed by measuring scotopic a-wave (rod photoreceptor function) and scotopic b-wave (inner retinal neuron function) recordings. The results indicate no significant difference between WT and IR KO retinas at 1 and 3 months of age (data not shown). The cone-driven photopic ERG b-wave recordings showed no abnormal signaling of cones to the inner retina at 1 and 3 months of age (data not shown). However, by 6 months of age, cone function was significantly reduced in IR KO mice compared with WT mice (Figure 4C). At this time point, we did not observe any significant differences between rod ERG (scotopic a-wave and scotopic b-wave amplitudes) from WT and IR KO mice (data not shown). These observations suggest that loss of cone IR has no effect on rod function, further attesting to the specific conditional deletion of IR in cones.

### Morphological characteristics of cone-IR KO mice

**Figure 5:**
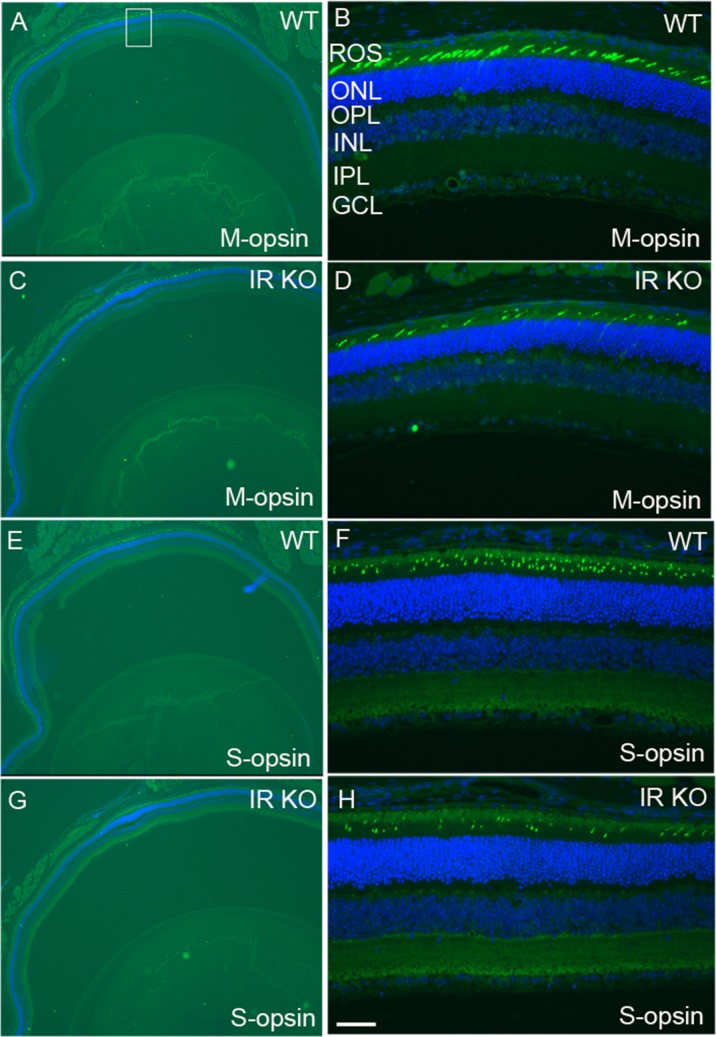
Loss of M- and S-cones in IR KO mice Retinal sections of WT **A., E.** and IR KO **C.,G.** mice at 6 months of age were stained for M-cone **A., B., C., D.** and S-cone **E.,F.,G.,H.** opsin, and nuclei were stained with DAPI. A region of the retina (square box) was photographed at 20X to show various layers **B., D., F., H.** ROS, rod outer segments; ONL, outer nuclear layer; OPL, outer plexiform layer; INL, inner nuclear layer; IPL, inner plexiform layer; GCL, ganglion cell layer. Scale bar 50 μm.

**Figure 6:**
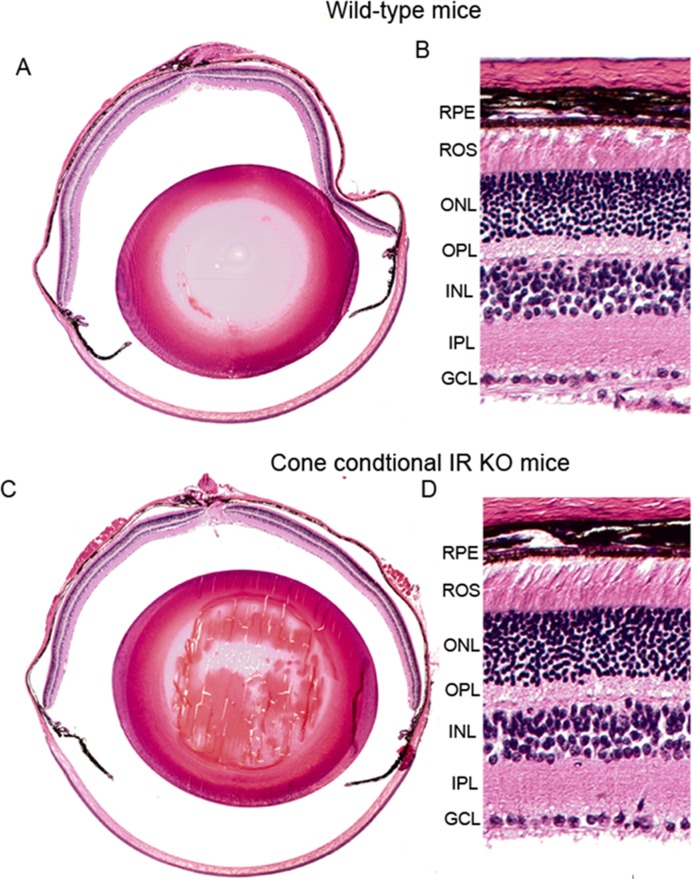
Morphology of cone-specific IR KO retina Morphological examination of retinas from six-month-old WT and IR KO mice. Five-micrometer-thick sections of retinas were cut along the vertical meridian and stained with hematoxylin and eosin **A., C.**. A region of the retina near the optic nerve head was enlarged for fine details from WT **B.** and IR KO retina **D.**. RPE, retinal pigment epithelium; ROS, rod outer segments; ONL, outer nuclear layer; OPL, outer plexiform layer; INL, inner nuclear layer; IPL, inner plexiform layer; GCL, ganglion cell layer.

We labeled cone outer segments as a function of cone cell viability. Lectin cytochemical analysis was performed using peanut agglutinin (PNA) on retinal flat mounts. Fluorescence microscopic analysis of WT and IR KO retinal flat mounts indicated a normal cone density at 1 and 3 months of age (data not shown). However, by 6 months, loss of cones appeared in IR KO retinas as patchy areas lacking cones, compared with WT retinas (Figure 4D). Retinal sections from 6-month-old WT and IR KO mice were examined by fluorescent microscopy with antibodies against M-cone opsin (Figure 5A-5D) and S-cone opsin (Figure 5E-5H) to positively identify cones. The results revealed that IR signaling is essential to cone survival, as ablation of IR results in the loss of both M- and S-cones. The morphology and structural integrity of the retinas from WT and IR KO mice were indistinguishable at 6 months of age (Figure 6). These observations suggest that the loss of cone photoreceptors in IR KO mice produces no structural changes in the overall morphology of the retina.

### Generation of cone conditional PTP1B knockout mice

**Figure 7:**
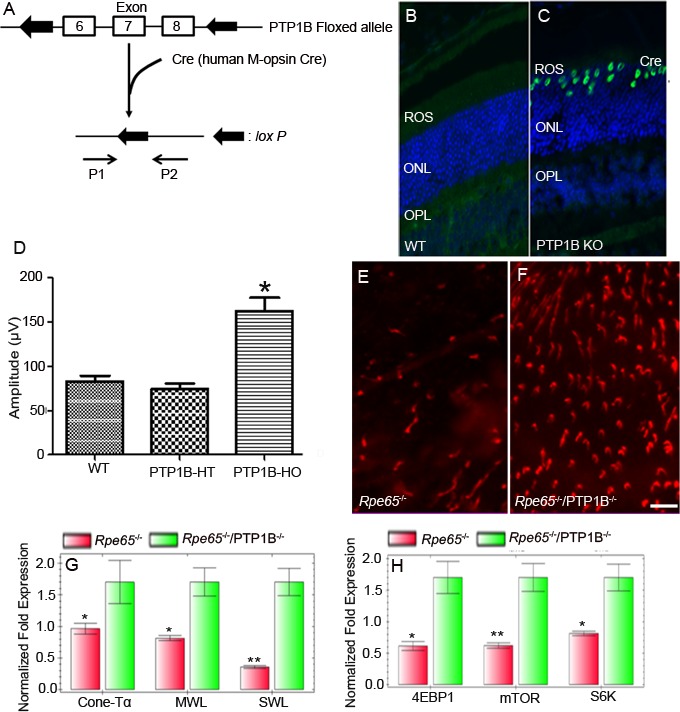
Cone-specific deletion of the PTP1B delayed cone degeneration in *Rpe65^−/−^* mice Schematic diagram of *loxP* floxed PTP1B loci **A.**. Cone photoreceptor-specific PTP1B KO mice were generated by breeding mice with a floxed PTP1B with mice that express Cre recombinase under the control of human M-opsin promoter. Primer pairs P1 and P2 were used to identify the wild-type and floxed PTP1B alleles. Immunohistochemical analysis of Cre recombinase in wild-type **B.** and PTP1B KO **C.** retinas. ROS, rod outer segments; ONL, outer nuclear layer; OPL, outer plexiform layer. Photopic b-wave electroretinographic analysis of WT, PTP1B heterozygous (PTP1B-HT) and PTP1B homozygous (PTP1B-HO) mice at 12 months of age **D.**. Values are means ± *SEM*. * *P* < 0.001 (*n* = 12). Retinal flat mounts from 5-week-old Rpe65 KO **E.** and Rpe65/cone-PTP1B double KO mice **F.** were stained with PNA. Scale bar 100 μm. Real-time PCR showing less expression of **G.** cone-specific markers (cone transducin α-subunit [GNA2], MWL-opsin, and SWL-opsin) and proteins involved in the mTOR pathway **H.** 4EBP1, mTOR, and S6K (p70S6K) in *Rpe65^−/−^* mice than in *Rpe65*/cone-PTP1B^−/−^ double KO mice. Data are means + *SD* (*n* = 3). *p < 0.05; **p < 0.001.

To generate cone conditional PTP1B knockout mice, M-opsin (red/green photopigment) promoter-driven Cre-recombinase mice were mated with mice expressing floxed PTP1B allele. Genomic DNA was extricated from tail biopsy, and cone-specific PTP1B knockout mice were identified by PCR using the primers described earlier [18, 31]. We, then, examined the cellular localization of Cre as a surrogate marker for PTP1B deletion in wild-type and cone-IR knockout littermates by immunofluorescence. Retinal sections prepared from wild-type and cone-PTP1B knockout mice were stained with anti-Cre antibody to localize Cre expression. Our results showed a nuclear localization of Cre in cone PTP1B knockout mice, but WT mice also showed Cre expression (Figure 7B-7C).

### Structural and functional characterization of cone conditional PTP1B knockout mice

The cone conditional PTP1B knockout mice were examined for function at 3, 6, and 12 months of age. At 3 and 6 months of age, there are no significant differences in either scotopic a- or scotopic b- wave amplitudes from wild-type, heterozygous PTP1B, or homozygous PTP1B knockout mice (data not shown). However, at 12 months of age, homozygous PTP1B knockout mice displayed a significantly higher photopic b-wave amplitude than wild-type and heterozygous PTP1B mice (Figure 7D). The increased cone function in 12-month-old PTP1B knockout mice was no different from the normal cone function that we observed in 3-month-old mice. These observations suggest that cone function decreases with age, and the absence of PTP1B protects against this loss. Consistent with the observed function, retinal flat mounts showed slightly increased cone density in PTP1B knockout mice compared with wild-type mice (data not shown).

### Loss of PTP1B in Rpe65^−/−^ cones delayed the death of cone photoreceptors

We previously reported increased PTP1B activity in *Rpe65*^−/−^ mouse retinas [22]. We also found that systemic administration of insulin to *Rpe65*^−/−^ mice delayed the death of cone photoreceptors (Figure 3). These observations suggest that increased PTP1B activity results in the inactivation of IR signaling, producing cone degeneration in this mouse model. It is technically difficult to measure PTP1B activity in < 5% cones, when rods comprise more than 95% of a WT mouse retina. To test our hypothesis that down-regulation of IR signaling in cones is due to increased PTP1B activity, we generated an *Rpe65*/cone-specific PTP1B double knockout mouse. Retinal flat mounts were prepared from 5-week-old *Rpe65* and *Rpe65/PTP1B* double knockout mice. We examined the cone density using PNA stain. The double knockout mice exhibited delayed death of cone photoreceptors compared with *Rpe65^−/−^* mice (Figure 7E-7F). Real-time PCR analysis was performed to confirm the expression levels of a subset of cone-specific photoreceptor genes using primers described in Table 1. The mRNA levels of these genes were normalized against 18s rRNA levels. The expression levels of cone photoreceptor-specific markers GNA2 (cone Tα), MWL-opsin, and SWL-opsin were significantly lower in the *Rpe65*^−/−^ retina than in the *Rpe65*^−/−^/cone-PTP1B^−/−^ double knockout retina (Figure 7G). The mammalian target of rapamycin (mTOR) pathway was previously shown to be downregulated in cones of RP mouse models [34]. The expression levels of mTOR pathway proteins, mTOR, 4EBP1, and p70S6K, were significantly lower in *Rpe65*^−/−^ retina than in *Rpe65*^−/−^/cone-PTP1B^−/−^ double knockout retina (Figure 7H). These observations suggest that the activation of IR signaling pathway due to the lack of PTP1B delays the death of cones in the *Rpe65*^−/−^ mouse retina. Thus, activated PTP1B may play a major role in cone survival; inhibiting its activity may promote cone viability.

**Table 1:** Real-time PCR primers used to measure gene expression

Gene	Forward Primer	Reverse Primer
PTP1B	ACCTGTGGGGATGAAGACAG	ATGCACACATTGACCAGGAA
Grb14	TTTCTTGGTACGGGATAGTCAGA	CAGCTGGATGAGGTCTGTGA
Src	TACCGTATGTCCCACATCCA	CCAGTTTCTCGTGCCTCAGT
mTOR	CGGTTTGGTGAAACCAGAAG	GTGAGATGTTGCCTGCTTGA
P70S6 Kinase (S6K)	CTCAGTGAAAGTGCCAACCA	CGCTCACTGTCACATCCATC
4EBP1	GGGGACTACAGCACCACTCC	ATCGCTGGTAGGGCTAGTGA
Rhodopsin	CAAGAATCCACTGGGAGATGA	GTGTGTGGGGACAGGAGACT
Sin	CTTTCCCAGTGGATCCTGAA	AAAGAGACATGGTGGCTGGT
SHP2	TATTCCTTGGTGGACCAGACA	GTGGGCTCATCTGAAACTCC
Cone Tα	GCATCAGTGCTGAGGACAAA	CTAGGCACTCTTCGGGTGAG
MWL-opsin	CTCTGCTACCTCCAAGTGTGG	AAGTATAGGGTCCCCAGCAGA
SWL-opsin	TGTACATGGTCAACAATCGGA	ACACCATCTCCAGAATGCAAG
18S RNA	TTTGTTGGTTTTCGGAACTGA	CGTTTATGGTCGGAACTACGA

### Expression of Src protein activators in the retina

Our earlier studies suggest that Grb14 phosphorylation is specific to the retina and is mediated by the non-receptor tyrosine kinase Src [22]. We previously reported that phosphorylated Grb14 inhibits the activity of PTP1B *in vivo* [22]. In *Rpe65*^−/−^ mice, we did not observe Src activation; this lack, in turn, impedes Grb14 phosphorylation [22]. In *Rpe65*^−/−^ mice, the opsin is properly localized to rod outer segments, but they do not photobleach, due to the deficiency of chromophore 11-*cis* retinal [35]. This results in the inactivation of IR signaling due to increased PTP1B activity in *Rpe65*^−/−^ mice [18], which may trigger cone degeneration [25]. Our hypothesis is that if Src is activated independent of rhodopsin activation, cone degeneration in *Rpe65*^−/−^ mice could be rescued.

**Figure 8:**
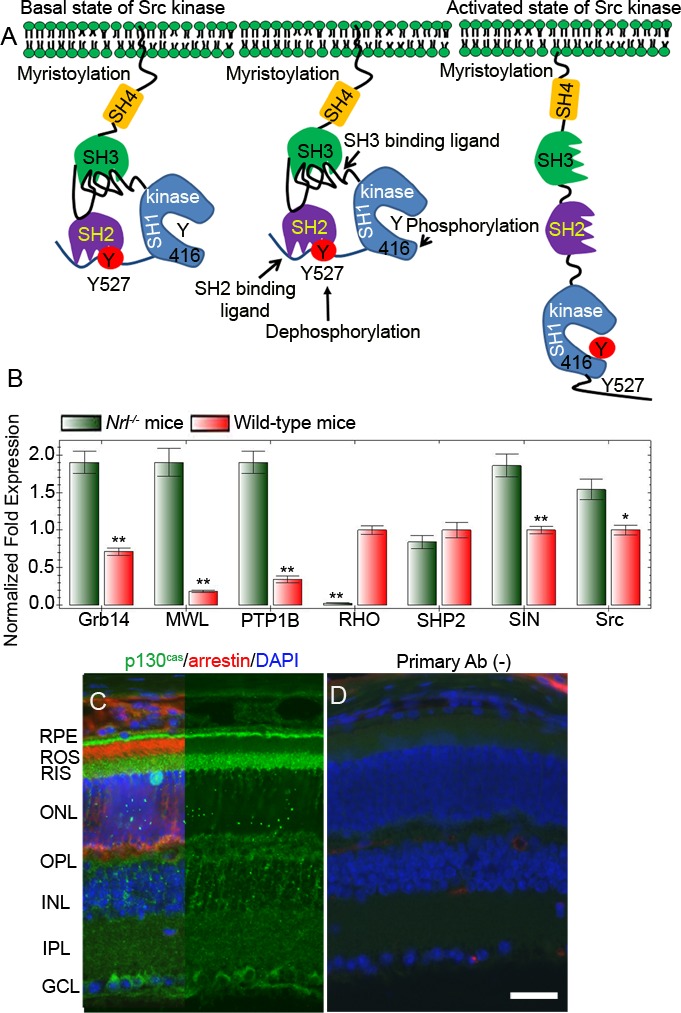
Mechanism of activation of Src Inactivation of Src through phosphorylation of Tyr527 and binding to its own SH2 domain **A.**. Activation of Src through dephosphorylation of Tyr527 or through binding of a proline-rich sequence containing proteins to SH3 domain **A.**. Real-time PCR showing the expression of Grb14, PTP1B, SHP2, Sin, Src, RHO (rod cell marker rhodopsin), and MWL (cone cell marker, medium wavelength cone opsin) in wild-type and *Nrl^−/−^* mouse retinas **B.**. Data are means + *SD* (*n* = 3), **p < 0.001; *p < 0.05. Light-adapted mouse retinal section was stained with p130^cas^ (green), arrestin (red), and DAPI (blue) **C.**. Panel D represents the omission of primary antibody **D.**. RPE, retinal pigment epithelium; ROS, rod outer segments; RIS, rod inner segment; ONL, outer nuclear layer; OPL, outer plexiform layer; INL, inner nuclear layer; IPL, inner plexiform layer; GCL, ganglion cell layer.

The proto-oncogene Src translates a tyrosine kinase and is commonly called non-receptor tyrosine kinase (NRTK). To date, there are nine known NTRKs, commonly referred to Src family kinases [36, 37]. The protein is organized into four distinct domains; an N-terminal SH4 domain contains a 14-carbon myristoyl group [38]. The co-translational myristoylation reaction that occurs on the N-terminal glycine is catalyzed by the enzyme N-myristoyltransferase [39]. This modification facilitates the membrane binding and subsequent activation of Src [38]. Src has also a Src-homology 3 (SH3) domain, capable of binding to proteins with proline rich-sequences, and a Src-homology (SH2) domain in the middle of the protein, capable of binding to phosphotyrosine residue, followed by an SH1 tyrosine kinase domain (Figure 8A). Near the C-terminus, an inhibitory tyrosine residue 527 undergoes phosphorylation by c-Src kinase [40]. The phospho tyrosine-527 (Y527) residue docks into its own SH2 domain and Src thus becomes inactive or is in a basal state. This configuration decreases the interaction between the SH3 domain and polyproline site (Figure 8A). In Src, the auto-inhibition of the kinase domain is performed by the SH2 and SH3 domains. Multiple mechanisms of cSrc activation have been reported. These include 1) the involvement of a phosphatase in the dephosphorylation of Y527 residue, 2) a competitive phosphotyrosine residue binding to the SH2 domain, and 3) competitive occupancy of the SH3 domain with a polyproline binding site.

We examined the effect of two Src activator proteins, Sin and p130^cas^, on Src activation and Grb14 phosphorylation. The novel Src kinase activator, Sin (Src interacting or signal integrating protein), binds to the SH3 domain and is expressed in the brain, thymus, and skeletal muscle [41]. However, the retina has not been examined for Sin protein expression. Since we do not have a Sin-specific antibody, real-time PCR using the primers described in Table 1 was used to examine the expression levels of Sin and other gene products (Grb14, PTP1B, SHP2, and Src) in wild-type and cone-dominant *Nrl^−/−^* mouse retinas. We used rhodopsin (RHO) and cone opsin (MWL) as rod and cone cell markers, respectively. Our results revealed that the expression levels of Grb14, PTP1B, Sin, and Src are significantly higher in cone-dominant retina than in the wild-type retina (Figure 8B). p130^cas^ binds directly to both the SH2 and SH3 domains of c-Src and activates the Src kinase [42]. Immunohistochemical analyses of p130^cas^ in the retina showed a predominant expression of this protein in the ganglion cell, outer plexiform, and photoreceptor layer (Figure 8C-8D). We also found the expression of p130^cas^ in cone-dominant *Nrl^−/−^* mouse retina (data not shown). These experiments suggest that Src regulatory proteins are present in the retina.

### Src phosphorylates Grb14 in the presence of Src-activator proteins *in vitro*

**Figure 9:**
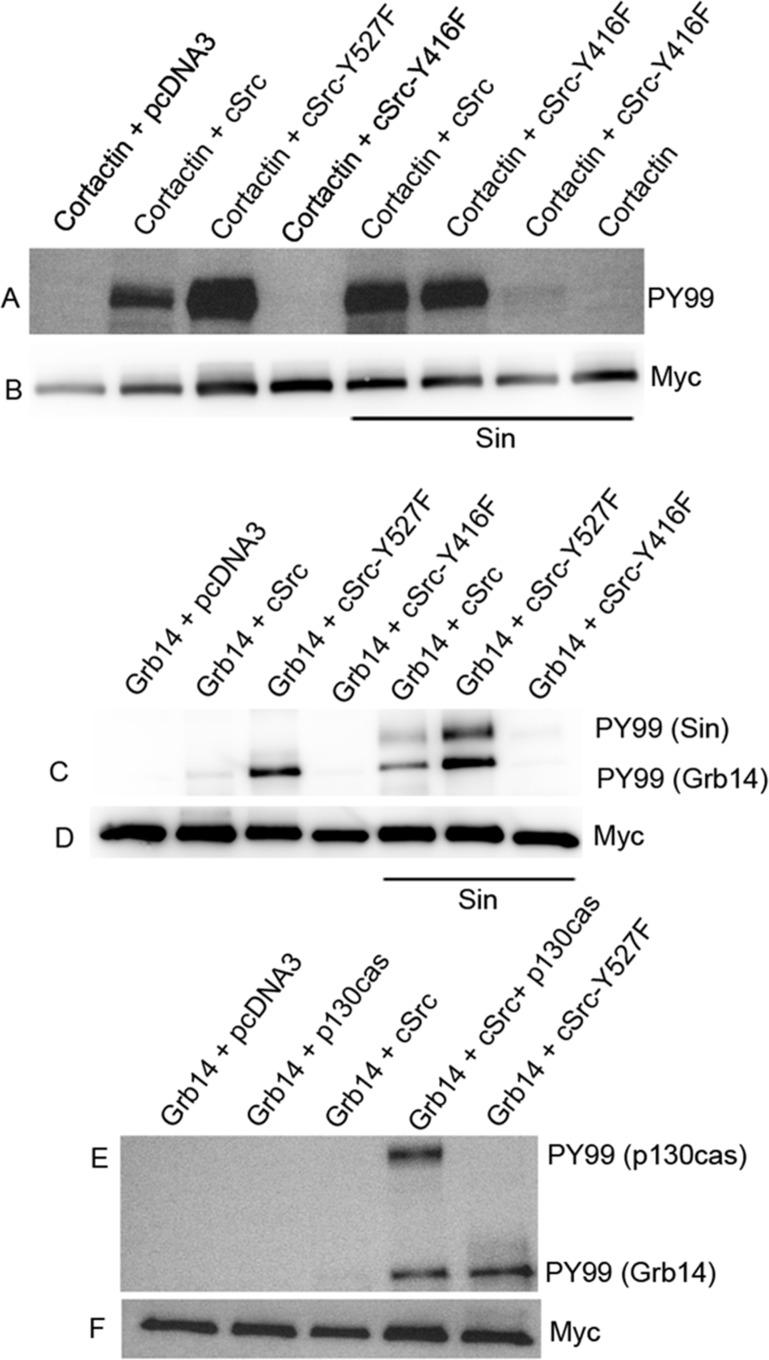
Activation of Src *in vitro* Myc-tagged cortactin or Grb14 were co-transfected or triple- transfected with the constructs pcDNA3, cSrc, cSrc-Y527F, cSrc-Y416F, Sin, or p130^cas^ in HEK-293T cells, as indicated in each figure. Myc immunoprecipitates were immunoblotted with PY99 antibody. Panel **A.-B.** represents experiments of cortactin phosphorylation by cSrc. Grb14 phosphorylation by cSrc was studied in the presence of Sin **C.-D.** and p130^cas^ **E.-F.**

To confirm the activation of Src, we tested the effect of Sin on one of the known cSrc substrates, cortactin [43]. We co-expressed Myc-tagged cortactin with pcDNA3 (control), cSrc, constitutively active mutant Src (Y527F), kinase dead mutant of cSrc (Y416F), or triple transfection (cortactin + Sin + WT cSrc, constitutively active cSrc, or kinase dead mutant cSrc) in HEK-293T cells. To examine the phosphorylation of cortactin, Myc-tagged proteins were immunoprecipitated with anti-Myc antibody, followed by immunoblot analysis with anti-PY99 antibody. The results indicate the phosphorylation of cortactin by WT cSrc. This phosphorylation was enhanced in the presence of Sin, suggesting the activation of cSrc by Sin (Figure 9A and 9B). The constitutively active mutant cSrc (Y527F) does not require Sin, as this mutant cSrc is able to phosphorylate cortactin, which is higher than in the presence of Sin (Figure 9A). The kinase dead mutant cSrc (Y416F) failed to phosphorylate cortactin in the presence and absence of Sin (Figure 9A). These experiments suggest that Sin activates cSrc *in vitro*.

We then examined the phosphorylation of Grb14 using the same constructs described in the cortactin experiment. Myc-tagged Grb14 expressed in HEK-293T cells was immunoprecipitated with anti-Myc antibody, and anti-PY99 antibody was used to probe the immunoblots. The results show that wild-type (WT) cSrc did not phosphorylate Grb14. However, the constitutively active mutant cSrc (Y527F) and wild-type cSrc were able to phosphorylate Grb14 in the presence of Sin (Figure 9C and 9D). It is interesting to note that the activated form of cSrc is also able to phosphorylate Sin protein (Figure 9C). These findings suggest that Grb14 phosphorylation can occur when we activate cSrc *in vitro*.

We also tested the effect of cSrc-mediated phosphorylation of Grb14 in the presence of p130^cas^ in HEK-293T cells. We found that cSrc-mediated the phosphorylation of Grb14 only in the presence of p130^cas^ (Figure 9E and 9F). The activated form of cSrc is also able to phosphorylate p130^cas^ protein (Figure 9E). Together, these findings suggest that Grb14 phosphorylation can be achieved by activating Src in the absence of rhodopsin signaling.

### Src activator sin delayed the death of cones in Rpe65^−/−^ mice

**Figure 10:**
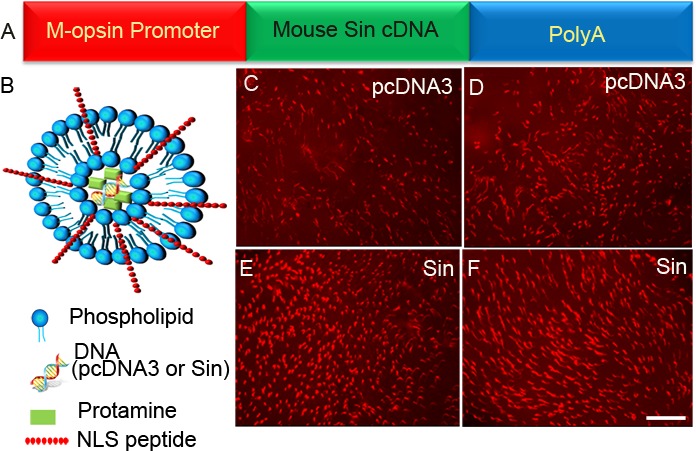
Sin delayed the death of cones in *Rpe65^−/−^* mice Sin cDNA was cloned under the control of a 2.1 kb human M-opsin promoter **A.** Control pcDNA3 vector **C.-D.** or Sin constructs **E.-F.** were complexed with LPD nanoparticles **B.** and injected subretinally into P5 *Rpe65^−/−^* mice. Flat mounts were prepared at P30 and stained with PNA. Scale bar 100 μm. NLS, nuclear localization signal peptide.

We hypothesized that Src-independent rhodopsin activation delays the death of cones in *Rpe65*^−/−^ mice. We cloned the cDNA encoding Sin under the control of a 2.1 kb human M-opsin (red/green pigment) promoter [44]. The control (pcDNA3) and Sin plasmid DNAs were complexed with lipid nanoparticles [45] and injected subretinally into P5 *Rpe65*^−/−^ mice. Flat mounts were prepared at P30 and stained with PNA. We found a greater protection of cone cell death in Sin-treated retinas than in control retinas (Figure 10). These observations suggest that activation of Src by Sin may promote cone viability. This protective effect may occur through the activation of insulin receptor signaling *via* phosphorylation of Grb14 by Src and subsequent inhibition of PTP1B by phosphorylated Grb14.

### Loss of Src activation and Grb14 phosphorylation in VPP-transgenic mice

**Figure 11:**
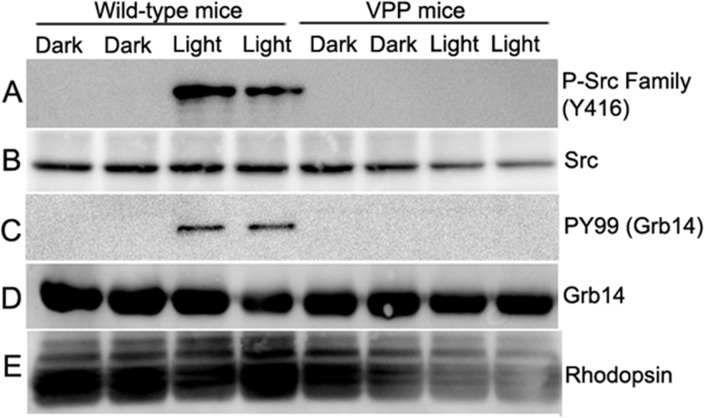
Loss of Src activation and Grb14 phosphorylation in VPP-transgenic mice Retinal lysates from two dark- and light-adapted WT and VPP transgenic mice were immunoblotted with anti-phospho-Src family (Y416) **A.**, anti-Src **B.**, and anti-rhodopsin **E.** antibodies. Grb14 immunoprecipitates from dark- and light-adapted WT and VPP mice were immunoblotted with anti-PY99 **C.** and reprobed with anti-Grb14 **D.** antibodies.

*Rpe65* gene mutations have been linked to recessive RP (~2% of cases) and Leber's Congenital Amaurosis (LCA, ~ 6% of cases) [46]. Transgenic VPP mutant mice have been generated by substituting valine 20 with glycine, proline 23 with histidine, and proline 27 with lysine in the opsin gene, which resembles one form of human autosomal dominant RP with progressive rod degeneration [47]. We previously reported increased PTP1B activity in rhodopsin mutant VPP-transgenic mice compared with wild-type mice [18]. To determine whether the increased PTP1B activity in VPP-transgenic mouse retina is due to reduced Src activation and subsequent Grb14 phosphorylation, Grb14 immunoprecipitates were prepared from retinal lysates from dark- and light-adapted WT and VPP mice probed with PY-99 antibody to detect Grb14 phosphorylation. These retinal lysates were also probed with phospho-Src family (Y416) antibody to detect phosphorylation on cSrc. To ensure an equal amount of protein in all of these experiments, we performed immunoblots with anti-Grb14, anti-Src, and anti-rhodopsin antibodies. Our results show that wild-type mice undergo a light-dependent phosphorylation of Src and Grb14 (Figure 11). In VPP-transgenic mouse retinas, we failed to observe phosphorylation of Src and Grb14 (Figure 11). These results suggest that inactivation of insulin receptor signaling could be a common occurrence, irrespective of recessive or dominant gene mutations in RP.

## DISCUSSION

Our earlier studies show that conditional ablation of the IR in rod photoreceptor cells caused light stress-triggered photoreceptor degeneration [32]. However, in the current study, deletion of the IR only in cones led to cone degeneration without stress (Figure 4 and 5). Our recently published results show that the light-dependent IR signaling pathway that we described in rods is also highly conserved in cones (Figure 2). We recently observed that reduced photobleaching in cones, such as occurs in *Rpe65^−/−^* mice, leads to cone degeneration, which we can delay by systemic administration of insulin. In *Rpe65^−/−^* mice, we confirmed the following: 1) rhodopsin does not photobleach [35], 2) light does not activate the IR [14], 3) Src is not activated [22], 4) Grb14 is not phosphorylated [22], and 5) PTP1B enzymatic activity is increased [18]. These observations led to the hypothesis that, in the absence of cone-opsin bleaching, the normal light-driven activation of IR signaling in cones does not occur, resulting in cone degeneration similar to what was seen in the mice conditionally deleted of the cone IR. We have also generated cone-specific IR/IGF-1R double knockout mice, and the phenotype is identical to IR knockout mice (data not shown). The functional role of IGF-1R in cones is not known, and we are currently investigating this role.

In the present study, we showed that ablation of PTP1B or activation of Src in *Rpe65*^−/−^ cones results in delayed cone cell death. Connie Cepko's laboratory found the same effect of systemic insulin administration of cone degeneration in the *rd1* mouse, but that prolonged treatment with insulin did not maintain the survival effect [34]. This could be due to the desensitization of the IR [48]. This suggests that insulin alone would not rescue cone degeneration. Targeting PTP1B or activating Src would be ideal to promote cone survival. We propose that the IR signaling pathway may be more important in cones than in rods, since anything that down-regulates this pathway results in cone degeneration. Since activation of this pathway delays cone death, there exists a promising window of opportunity for therapeutic intervention.

The current study has the limitation that we cannot measure retinal function in the *Rpe65*^−/−^ mice, as they do not photobleach [35]. We have also not examined the duration of cone survival in this model or the retinal distribution of cone opsins. These topics are important, but the objective of this study was to identify, validate, and provide the mechanistic aspects of the G-protein coupled receptor regulated tyrosine kinase signaling pathway for cone survival. Studies examining all of the aspects described above are underway in our laboratory. The present findings clearly suggest that PTP1B inhibitors and Src activators would be beneficial to promote cone survival. Findings from the two mouse models, *Rpe65*^−/−^ (recessive RP) and VPP transgenic mice (autosomal dominant RP), suggest that activation of PTP1B could be a common occurrence. The pathway that we identified may offer a novel and “universal” treatment for RP, irrespective of recessive or dominant gene mutations. This pathway may offer a novel therapeutic avenue to target the IR signaling pathway in RP mouse models, as opposed to fixing individual gene mutations.

Currently, how rhodopsin activation regulates the activity of Src *in vivo* is unknown. Src activation through beta-arrestin and GPCR has been reported outside of photoreceptors [49, 50]. Immunohistochemistry has shown the presence of beta arrestins in photoreceptors [51]. Light-dependent Src activation has been reported to promote its association with photoactivated rhodopsin and visual arrestin [52]. More in-depth studies are required to cognize the role of visual and non-visual arrestins in the modulation of Src kinase activity in the retina. Nevertheless, our results suggest that rhodopsin can initiate a non-canonical signaling pathway in addition to traditional phototransduction.

We previously reported that blocking the activity of Src kinase in *ex vivo* retinal explants failed to inhibit the phosphatase activity of PTP1B [22]. In *Rpe65*^−/−^ mice, opsin does not bleach. In VPP-transgenic mice, opsin photobleaching is normal, yet Src was not activated. These observations suggest that Src regulators, either phosphatase(s) that remove Y527 in Src or SH3- or SH2 binding ligands, could be under the control of a functional opsin molecule. The tyrosine phosphatase, Src-homology phosphotyrosyl phosphatase 2 (Shp2) has previously been shown to dephosphorylate Y527 residue in Src and its subsequent activation of Src [53]. We found the expression of Shp2 in the retina (data not shown). Further functional studies are required to confirm the regulation of Shp2 in the activation of Src in the retina *in vivo.*

Physiologically, the myristoylated Src anchors to the membrane, where PTP1α dephosphorylates the Y527 residue and Src thereby becomes active [54]. It has also been shown that deletion of tyrosine phosphatase PTP1α leads to a constitutive inactivation of Src kinase [54]. Thus, protein myristoylation may play an important role in Src activation in the retina. Our earlier studies revealed the expression of N-myristoyltransferase, which catalyzes the myristoylation reaction, in the retina [55, 56]. Understanding the Src regulators in various mouse models of disease may provide a window of opportunity to promote cone survival in retinal degenerative diseases.

## MATERIALS AND METHODS

### Materials

Purified mouse monoclonal anti-PI-3-P antibody was obtained from Echelon Biosciences, Inc. (Salt Lake City, UT). Polyclonal anti-Src and anti-Src family (Y416) antibodies were obtained from Cell Signaling Technology (Beverly, MA). Rabbit polyclonal anti-red/green cone opsin (M-opsin), polyclonal blue cone opsin (S-opsin), and anti-p130case antibodies were purchased from Millipore (Billerica, MA). Mouse monoclonal anti-Cre antibody suitable for immunohistochemistry was purchased from Abcam (Cambridge, MA). DAPI stain used for nuclear staining and secondary antibodies was purchased from Invitrogen-Molecular Probes (Carlsbad, CA). Monoclonal anti-arrestin antibody was a kind gift from Dr. Paul Hargrave (University of Florida, Gainesville). All other reagents were of analytical grade and purchased from Sigma (St. Louis, MO).

### Animals

All animals were treated in accordance with the *ARVO Statement for the Use of Animals in Ophthalmic and Vision Research* and the *NIH Guide for the Care and Use of Laboratory Animals.* The protocols were approved by the IACUC at the University of Oklahoma Health Sciences Center. Animals were born and raised in our vivarium and kept under dim cyclic light (40-60 lux, 12 h light/dark cycle). Floxed insulin receptor [32] and double floxed IR and IGF-IR [57] mice were kindly provided by Dr. Ronald C. Kahn (Joslin Diabetes Center, Harvard Medical School). The retinal pigment epithelium 65 (*Rpe65*) knockout mice were kindly provided by Dr. Jing-Xing Ma (University of Oklahoma Health Sciences Center, Oklahoma City). Floxed PTP1B mice [18] were kindly provided by Dr. Benjamin G. Neel (Ontario Cancer Center, Canada). The cone-dominant neural retina leucine zipper (*Nrl*) knockout mice were provided by Dr. Anand Swaroop (NEI, NIH, Bethesda, MD). A breeding colony of VPP (rhodopsin mutant) transgenic mice were obtained from Dr. Connie Cepko (Harvard Medical School). For experiments that required enucleating the eye or removing the retina, mice were killed by asphyxiation with CO_2_, followed by cervical dislocation. On the day of an experiment, animals were dark-adapted overnight. The next morning, at approximately 9:00 AM, half of the animals were exposed to normal fluorescent room light (300 lux) for 30 min [33] before the retinas were subjected to either biochemistry or immunohistochemistry.

### Plasmids and vectors

The cDNA encoding mouse Sin (Accession # NP_034242) was amplified from retina tissue by RT-PCR using sense (GAA TTC ACC ATG GCC ATT GCC ACG TCG GCC CAG) and antisense (GCGGCCGC TCA GGG GAG CAG ACC ATC GAG TAG) primers, and was cloned into pcDNA3 vector as an *EcoRI/Not1* fragment. The CMV promoter in the pcDNA3 vector was replaced with 2.1 kb human red-cone opsin promoter [44]. The woodchuck hepatitis virus post-transcriptional regulatory element (WPRE) sequence [44] was cloned into pcDNA3 downstream of Sin as a *Not1/XhoI* fragment. The pLNCX chick Src Y416F (Addgene plasmid # 13662) and pLNCX chick Src Y527F (Addgene plasmid # 13660) constructs were a gift from Joan Brugge. The pCMV5 mouse Src was a gift from Joan Brugge and Peter Howley (Addgene plasmid # 13663). The mouse p130^cas^ expression construct was obtained from Origene (Rockville, MD). The cortactin construct was a gift from Michel L. Tremblay (McGill University, Canada).

### Generation of Rpe65^−/−^/cone PTP1Bα^−/−^ double knockout mice

In the initial breeding protocol, *Rpe65^−/−^* mice were crossed with cone Cre^+/−^PTP1B^f/f^ (cone Cre-PTP1B knockout) mice. The resultant F1 generation yielded two genotypes (50% each), *Rpe65^+/−^*/cone Cre^−/−^PTP1B^w/f^ and *Rpe65^+/−^*/cone Cre^+/−^PTP1B^w/f^. In the next breeding, we crossed *Rpe65^+/−^*/cone Cre^+/−^PTP1B^w/f^ mice with *Rpe65^−/−^* mice. From the offspring, we selected the *Rpe65^−/−^*/cone Cre^+/−^PTP1B^w/f^ mice and mated them with Rpe65*^−/−^*/cone Cre^−/−^PTP1B^w/f^ mice. The resultant F3 generation yielded 6 genotypes: *Rpe65^−/−^*/cone Cre^−/−^PTP1B^f/f^ (12.5%), *Rpe65^−/−^*/cone Cre^−/−^PTP1B^w/f^ (25%), *Rpe65^−/−^*/cone Cre^−/−^PTP1B^w/w^ (12.5%), *Rpe65^−/−^*/cone Cre^+/−^PTP1B^f/f^ (12.5%), *Rpe65^−/−^*/cone Cre^+/−^PTP1B^w/f^ (25%), and *Rpe65^−/−^*/cone Cre^+/−^PTP1B^w/w^ (12.5%). Genotyping was performed by PCR analysis of genomic DNA extracted from tail snips. Each mouse was genotyped for cone opsin-Cre, PTP1B floxed allele, and *Rpe65.* For Cre genotype screening, a forward primer, TTG GTT CCC AGC AAA TCC CTC TGA, designed within the promoter DNA sequence and a reverse primer, GCC GCA TAA CCA GTG AAACAG CAT, designed within the Cre sequence were used to amplify the PCR product of 411 bp. To identify PTP1B^floxed^ mice, we used a sense primer, 5′-TGC TCA CTC ACC CTG CTA CAA-3′, and an antisense primer, 5′-GAA ATG GCT CAC TCC TAC TGG-3′, to amplify genomic DNA by PCR. The wild-type (WT) allele generates a 206-bp product, and the floxed allele generates a 327-bp product. For *Rpe65* genotyping, we used three primers: 5′- GGG AAC TTC CTG ACT AGG GGA GG -3′, 5′- GAT GTG GGC CAG GGC TCT TTG AAG -3′, and 5′- CCC AAT AGT CTA GTA ATC ACA GAT G-3′. The reaction generates a 546-bp wild-type and/or a 459-bp mutant allele fragment.

### Preparation of liposome protamine/DNA lipoplexes (LPD)

LPD nanoparticles were prepared according to the method reported previously [45]. First, the liposomes consisting of DOTAP (1, 2-dioleoyl-3-trimethylammonium-propane), DOPE (1, 2-dioleoyl-*sn*-glycero-3-phosphoethanolamine), and cholesterol (1:1:1 molar ratio; Avanti Polar Lipids, Inc., USA) were prepared by thin-film hydration. Second, protamine (1.25 mg/mL; Sigma-Aldrich Co. LLC, USA), nuclear localization peptide from SV40 (0.5 mg/mL), and the plasmid DNA (pDNA) were mixed. The resultant protamine/pDNA complex was allowed to stand at room temperature for 20 min. The above liposome was then added into the protamine/ pDNA complex, followed by vortexing. The resultant mixture was kept at room temperature for another 20 min to form liposome/protamine/DNA lipoplexes.

### Phosphoinositide 3-kinase (PI3K) assay

Enzyme assays were carried out as previously described [58]. Briefly, assays were performed directly on IR immunoprecipitates of retinal lysates prepared from light- and dark-adapted *Nrl^−/−^* mouse retinas in 50 μl of reaction mixture containing 0.2 mg/ml PI-4,5-P_2_, 50 μM ATP, 10 μCi [γ;^32^P]ATP, 5 mM MgCl_2_, and 10 mM HEPES buffer (pH 7.5). The reactions were carried out for 30 min at room temperature and were stopped by the addition of 100 μl of 1 N HCl, followed by 200 μl of chloroform/methanol (1/1, v/v). Lipids were extracted and resolved on oxalate-coated TLC plates (silica gel 60) with a solvent system of 2-propanol/2 M acetic acid (65/35, v/v). The plates were coated in 1% (w/v) potassium oxalate in 50% (v/v) methanol and were then baked in an oven at 100°C for 1 h before use. TLC plates were exposed to X-ray film overnight at −70°C. Radioactive lipids were scraped and quantified by liquid scintillation counting.

### Immunostaining of retinal whole-mounts

Eyes were enucleated and placed in cold Hanks’ balanced salt solution buffered with 25 mM HEPES (pH 7.2), after which the corneas and lenses were removed and retinas were carefully isolated. Relaxing cuts were made in the retinal margins, and the whole retina was flattened onto a black filter membrane. Whole-mounted retinas were fixed in 4% paraformaldehyde in PBS at 4°C for 2 h and were rinsed in PBS. Non-specific labeling was blocked using 10% horse serum in PBS. Whole-mounts were incubated in a combination of biotinylated PNA (1:500) overnight at 4°C. Streptavidin conjugated to Texas red (1:250) was used to visualize peanut agglutinin (PNA) labeling. Labeling in retinal whole-mounts was imaged using either a Nikon Eclipse E800 (Tokyo, Japan) or an Olympus IX70 (Olympus USA, Center Valley, Pennsylvania) epifluorescence microscope.

### Preparation of tissue for paraffin sectioning using prefer as a fixative

Mice were euthanized by CO_2_ asphyxiation. The eyeballs were placed in Prefer solution (Anatech Ltd, Battle Creek, MI) for 15 min at room temperature, followed by 70% ethanol overnight. The tissue was paraffin-embedded, and 5-μm thick sections were cut and mounted onto slides. Sections were deparaffinized in 2-3 changes of xylene (10 minutes each) and were hydrated in 2 changes of 100% ethanol for 3 min each, 95% and 80% ethanol for 1 min each, and were then rinsed in distilled water. The slides were then subjected to antigen retrieval, boiled in 10 mM sodium citrate buffer pH 6.0, at a sub-boiling temperature for 10 min, and were cooled for 30 min. The slides were washed three times in 1X PBS containing 0.1% Triton-X 100, and were blocked with horse serum for 1 h. Primary antibody was added overnight at 4°C. For fluorescent detection, slides were incubated with a mixture of Texas-red-anti-mouse and FITC-anti-rabbit antibodies (Vector Laboratories, Burlingame, CA), each diluted 1:200 in PBS with 10% horse serum. Following incubation for 1 h at room temperature, the slides were washed with PBS and cover-slipped in 50% glycerol in PBS. Antibody-labeled complexes were examined on a Nikon Eclipse E800 microscope equipped with a digital camera. Images were captured using Metamorph (Universal Imaging, West Chester, PA) image analysis software. All images were captured using identical microscope and camera settings.

### Statistical analysis

One-way ANOVA and post-hoc statistical analysis using Bonferroni's pairwise comparisons were used to determine statistical significance (*p* < 0.05).
